# *Vernonia condensata* Baker (Asteraceae): A Promising Source of Antioxidants

**DOI:** 10.1155/2013/698018

**Published:** 2013-12-31

**Authors:** Jucélia Barbosa da Silva, Vanessa dos Santos Temponi, Carolina Miranda Gasparetto, Rodrigo Luiz Fabri, Danielle Maria de Oliveira Aragão, Nícolas de Castro Campos Pinto, Antônia Ribeiro, Elita Scio, Glauciemar Del-Vechio-Vieira, Orlando Vieira de Sousa, Maria Silvana Alves

**Affiliations:** ^1^Graduate Program in Pharmaceutical Sciences, Federal University of Juiz de Fora, Campus Universitário, 36036-330 Juiz de Fora, MG, Brazil; ^2^Faculty of Pharmacy, Federal University of Juiz de Fora, Campus Universitário, 36036-330 Juiz de Fora, MG, Brazil; ^3^Department of Biochemistry, Institute of Biological Sciences, Federal University of Juiz de Fora, Campus Universitário, 36036-330 Juiz de Fora, MG, Brazil

## Abstract

The present study evaluated the antioxidant potential of *Vernonia condensata* Baker (Asteraceae). Dried and powdered leaves were exhaustively extracted with ethanol by static maceration followed by partition to obtain the hexane, dichloromethane, ethyl acetate, and butanol fractions. Total phenols and flavonoids contents were determined through spectrophotometry and flavonoids were identified by HPLC-DAD system. The antioxidant activity was assessed by DPPH radical scavenging activity, TLC-bioautography, reducing power of Fe^+3^, phosphomolybdenum, and TBA assays. The total phenolic content and total flavonoids ranged from 0.19 to 23.11 g/100 g and from 0.13 to 4.10 g/100 g, respectively. The flavonoids apigenin and luteolin were identified in the ethyl acetate fraction. The IC_50_ of DPPH assay varied from 4.28 to 75.10 *µ*g/mL and TLC-bioautography detected the antioxidant compounds. The reducing power of Fe^+3^ was 19.98 to 336.48 **μ**g/mL, while the reaction with phosphomolybdenum ranged from 13.54% to 32.63% and 56.02% to 135.00% considering ascorbic acid and rutin as reference, respectively. At 30 mg/mL, the ethanolic extract and fractions revealed significant effect against lipid peroxidation. All these data sustain that *V. condensata* is an important and promising source of bioactive substances with antioxidant activity.

## 1. Introduction

Free radicals and oxidants play a dual role as both toxic and beneficial compounds, since they can be either harmful or helpful to the body [1]. These molecules are produced either from *in situ* normal cell metabolisms or from environmental sources including ionizing radiation, UV light, pesticides, alcohol, cigarette smoke, and oxygen shortage with the generation of reactive oxygen species (ROS) [1, 2]. ROS, as hydrogen peroxide (H_2_O_2_) and superoxide (O_2_^•−^), are produced by cellular reactions, including the iron-catalysed Fenton reaction, and by several enzymes as lipoxygenases, peroxidases, NADPH oxidase, and xanthine oxidase [1, 3]. Among the main cellular components susceptible to damage by free radicals, lipids (peroxidation of unsaturated fatty acids in membranes), proteins (denaturation), carbohydrates, and nucleic acids are highlighted [1, 4]. These damages have been implicated in the pathogenesis of many human diseases as neurodegenerative disorders like Alzheimer's disease, Parkinson's disease, multiple sclerosis, amyotrophic lateral sclerosis, memory loss, and depression; cardiovascular diseases like atherosclerosis, ischemic heart disease, cardiac hypertrophy, and hypertension; pulmonary disorders like inflammatory lung diseases such as asthma and chronic obstructive pulmonary disease; diseases associated with premature infants, including bronchopulmonary dysplasia, periventricular leukomalacia, intraventricular hemorrhage, retinopathy of prematurity, and necrotizing enterocolitis; autoimmune disease like rheumatoid arthritis; renal disorders like glomerulonephritis and tubulointerstitial nephritis, chronic renal failure, proteinuria, and uremia; gastrointestinal diseases like peptic ulcer, inflammatory bowel disease, and colitis; tumors and cancers like lung cancer, leukemia, breast, ovary, rectum cancers, and others; ageing process; diabetes; skin lesions; immunodepression; and liver disease, pancreatitis and infertility [1, 4, 5].

An update of the researches carried out in the last decades with this focus showed that the antioxidants of plant origin with properties against free-radicals could have great importance as therapeutic agents in several diseases related to oxidative stress, since these compounds are effective as radical scavengers and inhibitors of lipid peroxidation [5, 6]. In this context, the use of these natural products has proved to be clinically efficient and relatively less toxic than the existing drugs [5] and produce beneficial effects for human health through metabolic activities such as metabolic regulation, metabolic energy control, and activation/inactivation of biomolecules, signal transduction, cell exchange, endothelium-related vascular functions, and gene expression [7]. In addition, natural products used in food as flavoring and in medicinal mixtures, often contain high concentrations of phenolic compounds that have strong H-donating activity [8–10]. The major plant-derived phenolic antioxidants can be divided into four general groups as follows: phenolic acids, phenolic diterpenes, flavonoids, and volatile oils. Considering the mechanism of action of these substances, phenolic acids generally act as antioxidants by trapping free radicals and flavonoids can scavenge free radicals and chelate metals [7, 9]. Therefore, vegetal drugs or phytoconstituents with high concentration of phenolic substances may be used to prevent and/or treat oxidative stress.

In view of the global biodiversity, *Vernonia *represents a part of the large group of medicinal plants worldwide used, including Brazil [11]. This genus has approximately 1,500 species and has long been popularly used to treat several types of disorders including inflammation, malaria, fever, worms, pain, diuresis, cancer, abortion, and several gastrointestinal problems [12]. Under the pharmacological light, species of *Vernonia* has showed hypotensive [13], phototoxic, antibacterial and anti-inflammatory [14], immunomodulatory [15], and anti-histaminic effects [16]. These activities can be attributed to the presence of some reported special metabolites as glycosides, terpenes, steroids, and flavonoids [11, 15, 17, 18] represented by polysaccharides [15], vernolepin [16], hesperidin, 3′-methylhesperetin, homoesperetin-7-O-rutinoside, sitosterol, and stigmasterol [19].

Despite the great diversity of *Vernonia *species, only few scientific articles have been published, particularly about *Vernonia condensata *Baker (Asteraceae) [20–24], commonly known in Brazil as “figatil” or “necroton” and traditionally used as analgesic, anti-inflammatory, antithermal, antianemics, antibacterial, liver tonic, hepatoprotective, and antiulcerogenic agents [20]. Analgesic, anti-inflammatory and antiulcerogenic activities of *V. condensata*, as well as its toxicity had been investigated [20–24]. In these studies, vernonioside B2 demonstrated antinociceptive and anti-inflammatory effects [23]. Furthermore, saponins, tannins, alkaloids, phenolic compounds, and flavonoids were also detected in *V. condensata* [24].

Considering the medicinal uses, particularly its use in the treatment of disorders that involve the production of free radicals as inflammation, pain, cancer, abortion, and gastrointestinal disturbances, and the lack of scientific validation supported by experimental studies, the present investigation was designed to evaluate the antioxidant effect of the ethanol extract and fractions obtained from *V. condensata* leaves. In addition, total phenolic and flavonoid contents and chemical characterization were conducted in order to quantify and identify antioxidant constituents in this medicinal plant.

## 2. Materials and Methods

### 2.1. Plant Material

Specimens of *Vernonia condensata* Baker (Asteraceae) were cultivated at the Medicinal Garden of the Faculty of Pharmacy, Federal University of Juiz de Fora, Juiz de Fora city, Minas Gerais state, southeast region of Brazil. Mature leaves were collected from July to September 2010, and a voucher specimen (CESJ number 52943), identified by Dr. Fátima Regina Gonçalves Salimena, was deposited in the Herbarium Leopoldo Krieger of the Federal University of Juiz de Fora, Brazil. The leaves were dried at room temperature with forced ventilation for a loss of 90–96% humidity. After drying, all material was triturated by an industrial blender and pulverized using a tamise n° 18 for the extract preparation.

### 2.2. Extract Preparation

Dried and powdered mature leaves (465 g) were exhaustively extracted in 95% ethanol (2.5 L) by static maceration for 3 weeks at room temperature with renewal of solvent every 2 days. The ethanol extract was filtered and evaporated under a rotary evaporator at controlled temperature (50–60°C). This material was placed into a desiccator with silica to yield 27 g. The ethanol extract (EE) was suspended in water : ethanol (9 : 1) followed by liquid/liquid partition with increasing organic solvent polarity : hexane, dichloromethane, ethyl acetate, and butanol. After this procedure, hexane (HF), dichloromethane (DF), ethyl acetate (EF), and butanol (BF) fractions were obtained [25].

### 2.3. Chemicals and Reagents

Chemicals and reagents used in this study (and their sources) were as follows: DPPH, thiobarbituric acid, gallic acid, rutin, luteolin, and apigenin (Sigma Chemical Co, St. Louis, MI, USA); aluminum chloride, potassium ferrocyanide, dichloromethane, hexane, butanol, methanol, ethanol, pyridine, and sodium carbonate (Labsynth, Diadema, SP, Brazil) and Folin-Ciocalteu reagent, trichloroacetic acid, ascorbic acid, and acetonitrile (Cromoline Química Fina, Diadema, SP, Brazil). All the chemicals used including the solvents were of analytical grade.

### 2.4. DPPH Radical Scavenging Activity

DPPH was used for determination of free radical-scavenging activity [26]. Different concentrations of each sample [EE (20, 25, 30, 35, 40, 50, and 60 *μ*g/mL), HF (80, 100, 140, 150, and 160 *μ*g/mL), DF (5, 10, 20, 30, 50, 60, and 70 *μ*g/mL), EF (1, 3, 5, 10, 20, 25, and 30 *μ*g/mL), and BF (30, 40, 50, 60, and 70 *μ*g/mL)] were added, at an equal volume, to methanol solution of DPPH (0.03 mM). After 60 min at room temperature, the absorbance was recorded at 518 nm. The experiment was performed in triplicate. Rutin was used as standard control. IC_50_ values denote the concentration (*μ*g/mL) of sample, which is required to scavenge 50% of DPPH free radicals.

### 2.5. TLC-Bioautography Assay

20 *μ*L of each sample (EE, HF, DF, EF, and BF) (10 mg/mL) were applied on thin layer chromatography (TLC) (7 cm × 2 cm) of silica gel 60 F_254_ which was eluted with dichloromethane : methanol (5% or 15%) in duplicate [27]. The separated components were visualized under UV (254 and 366 nm) and visible light or spraying with specific reagent: vanillin sulfuric acid (general reagent), Dragendorff (alkaloids), Lierbermann-Burchard (terpenoids), ferric chloride solution (phenolic compounds), KOH 10% (coumarin), and NP/PEG (flavonoids). Furthermore, each sample on TLC was also sprayed with 2.5 mM DPPH (2,2-diphenyl-1-picrylhydrazyl) in ethanol and the presence of yellow stains was indicative of components with antioxidant activity.

### 2.6. Test of Iron Reducing Power

The reducing power of iron was determined using a serial dilution of the samples (250; 125; 62.50; 31.25; 15.62; and 7.81 *μ*g/mL for HF and DF, and 53.64; 28.82; 13.41; 6.70; 3.35; and 1.67 *μ*g/mL for EF, BF, and EE) with 2.5 mL of 0.2 mM phosphate buffer pH 6.6, and 2.5 mL of 1% potassium ferrocyanide [K_3_Fe(CN)_6_] [28]. The mixture was incubated at 50°C for 20 min. Five milliliters of this mixture received 2.5 mL of 10% trichloroacetic acid and after that, this solution was centrifuged at 3,000 g for 10 minutes. The supernatant was separated and mixed with 2.5 mL distilled water containing 0.5 mL 1% ferric chloride. The absorbance of this mixture was measured at 700 nm in triplicate. Ascorbic acid was used as reference material. The measurement was considered the possible antioxidant activity.

### 2.7. Test of Phosphomolybdenum Reducing Power

The assay was based on the reduction of molybdenum (VI) to molybdenum (V) which occurred in the presence of antioxidant substances with consequent formation of a green complex between phosphate/molybdenum (V) in acidic pH, which is spectrophotometrically determined at 695 nm [29]. The antioxidant activity was expressed as relative antioxidant activity (RAA%) of ascorbic acid and rutin.

### 2.8. Thiobarbituric Acid Method (TBA Method)

The TBA test determines the presence of malonaldehyde and other substances from lipid peroxidation [30], forming a colored complex which is determined by spectrophotometry at 535 nm. The concentration of TBA-Malondialdehyde (MDA) complex was established from the MDA standard curve using butanol as a blank. Analyses were performed in triplicate and BHT was used as reference.

### 2.9. Total Phenolic Determination

The total phenolic content was determined by Folin-Ciocalteu method [31] using gallic acid as reference standard (standard curve was prepared with concentrations 10 to 50 *μ*g/mL). The samples were oxidized with Folin-Ciocalteu reagent and the reaction was neutralized with sodium carbonate. The absorbance of the resulting blue color was measured at 765 nm after 60 min. The analyses were performed in triplicate and results were expressed as gram of gallic acid equivalent.

### 2.10. Total Flavonoids Determination

Aluminum chloride colorimetric method was used for total flavonoid determination [32] using rutin as standard. Each sample (0.4 mL) was separately mixed with 0.12 mL of acetic acid, 2 mL of pyridine : ethanol (2 : 8), 0.5 mL of 8% aluminum chloride, and 1.98 mL of distilled water and after that remained at room temperature for 30 min. The absorbance of the reaction mixture was measured at 420 nm with a double beam UV/Visible spectrophotometer. The calibration curve was prepared with rutin solutions in ethanol (2 to 30 *μ*g/mL) and results were expressed as gram of rutin equivalent.

### 2.11. High Pressure Liquid Chromatography (HPLC) Analysis

HPLC analysis was performed using an Agilent Technologies 1200 Series, with a PDA detector and an automatic injector. The column employed was a Zorbax SB-18; 250 × 4.6 mm, 5 *μ*m particle size. Mobile phase was composed by solvents A (water pH adjusted to 4.0 with H_3_PO_4_) and B (acetonitrile). The elution conditions applied were 0–30 min, 20% B isocratic. The mobile phase was returned to the original composition over the course of 30 min and additional 5 minutes were allowed for the column to re-equilibrate before injection of the next sample. The sample volume was 50 *μ*L at a concentration of 1 mg/mL, the flow rate of 0.6 mL/min, and the temperature was maintained at 25°C during the analysis. Detection was performed simultaneously at 210, 230, 254, 280, and 330 nm. Two pure standards, luteolin and apigenin, previously identified in *Vernonia* [33, 34], were used in this experiment as markers. For all experiments, EF and the standards were dissolved in methanol.

### 2.12. Statistical Analysis

Data were expressed as mean ± S.E.M. Statistical significance was determined by one-way analysis of variance followed by the Tukey test. *P* < 0.05 was considered significant.

## 3. Results

### 3.1. DPPH Radical Scavenging and Reducing Power Activities

Initially, TLC-bioautography method was performed to screen the antioxidant capacity of *V. condensata.* After separation on TLC plates, the components of EE, HF, DF, EF, and BF with radical scavenging activity were sprayed with DPPH reagent followed by UV and visible light detection. The presence of yellowish bands on the purple background color was considered as antioxidants. In addition, TLC plates with the same samples were also revealed with specific reagents and phenolic, flavonoids, and terpenoids compounds were detected.  Table 1 shows the scavenging effects obtained with samples on DPPH radical in the following order: EF > EE > BF > DF > HF. The IC_50_ values were statistically different (*P* < 0.05) that ranged from 18.44 ± 0.54 to 147.14 ± 0.40 *μ*g/mL (Table 1). HF and EE were more active to inhibit the DPPH radical with IC_50_ equal to 18.44 ± 0.54 and 35.44 ± 0.76 *μ*g/mL, respectively (Table 1). In addition, the ranking order for reducing power was EF > BF > EE > HF > DF. The iron reducing power produced IC_50_ values between 19.98 ± 0.42 and 336.48 ± 11.05 *μ*g/mL. As noted in the DPPH test, EF was more potent in convert Fe (+3) to Fe (+2) with IC_50_ of 19.98 ± 0.42 *μ*g/mL.

### 3.2. Phosphomolybdenum Assay

The phosphomolybdate method is also a quantitative assay, since the total antioxidant capacity is expressed as rutin or ascorbic acid equivalents. For rutin, the antioxidant capacity of samples of *V. condensata* was found to decrease in this order: HF < EE < BF < EF < DF (Table 3). The IC_50_ value of antioxidant capacity for the HF (56.02 ± 0.06%) was most pronounced (*P* < 0.05) than EE (94.87 ± 0.09%) and BF (105.00 ± 0.04%). The antioxidant activity with ascorbic acid also decreased at the same order as rutin: HF < EE < BF < EF < DF (Table 2). In this case, the IC_50_ values ranged from 13.54 ± 0.01 to 32.63 ± 0.01%.

### 3.3. Antioxidant Activity by TBA Method

The antioxidant activity was measured using thiobarbituric acid (TBA) during 5 days. In the last day, the MDA concentration declined in the homogenate treated with EE, HF, DF, EF, and BF at concentrations of 7.5, 15, and 30 mg/mL (Table 3). EF was the most active in inhibiting the formation of MDA in homogenate, specially at 30 mg/mL (0.18 ± 0.01 mmol/L).

### 3.4. Total Phenolic and Flavonoids Contents

Total phenolic content was estimated by using Folin-Ciocalteu reagent, expressed as gram of gallic acid equivalent, while flavonoid was quantified by aluminum chloride method and expressed as gram of rutin equivalent. In *V. condensata*, the total phenolic varied from 0.19 to 23.11 g/100 g and flavonoid ranged from 0.13 to 4.10 g/100 g (Table 4). In addition, this Table also showed that the EF exhibited the highest total phenolic (23.11 ± 0.90 g/100 g) and the highest amount of flavonoid contents (4.10 ± 0.03 g/100 g).

### 3.5. Analysis of the Ethyl Acetate Fraction by HPLC

**Figure 1:**
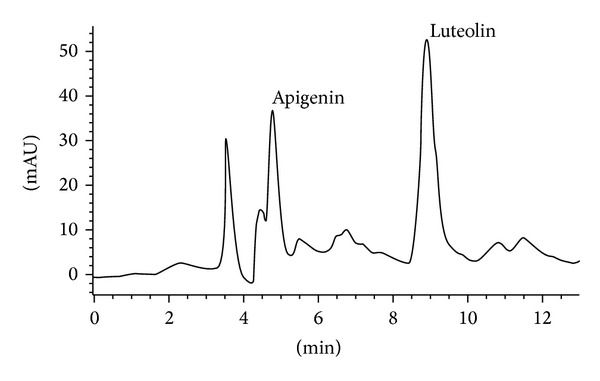
HPLC chromatogram of the ethyl acetate fraction of *Vernonia condensata*. The analysis was performed using a binary solvent system A (water pH adjusted to 4.0 with H_3_PO_4_), B (acetonitrile) in an isocratic run. The elution conditions applied were 0–30 min, 20% B. It was run at a flow rate of 0.6 mL/min over 30 minutes, with an injection volume (“loop”) of 50 *μ*L and UV detection was at 330 nm.

**Figure 2:**
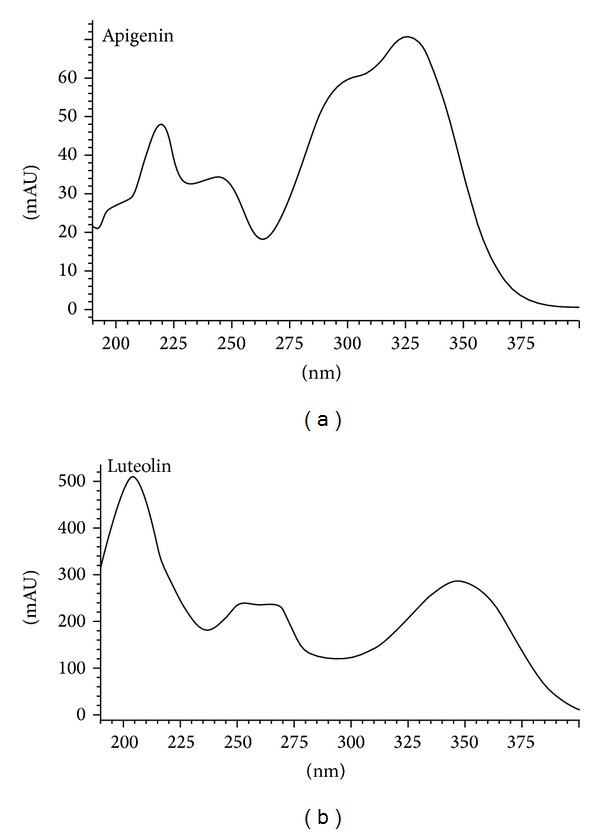
UV spectra of apigenin and luteolin found in the ethyl acetate fraction of *Vernonia condensata*.

Due to the higher content of flavonoids, EF was subjected to HPLC-DAD analysis. The elution conditions applied allowed the identification of the flavonoids apigenin (Rt = 4.72) and luteolin (Rt = 8.86). The chromatogram and the respective UV spectra are shown in Figures 1 and 2, respectively.

## 4. Discussion

Antioxidants are radical scavengers' substances that delay or inhibit oxidative damage by blocking the oxidizing chain reactions. These compounds prevent cell and tissue damages and can neutralize free radicals by donating electrons, ending the carbon-stealing reaction. In body and at low concentration, these molecules markedly delay or prevent the oxidation of an oxidizable substrate [1–3]. Consequently, the antioxidants play important roles in delaying the development of chronic diseases such as cardiovascular diseases, cancer, atherosclerosis, inflammatory bowel syndrome, and Alzheimer's diseases [4, 5]. In addition, the interest in natural products, specially phenolic compounds, has increased based on their capacity as antioxidants and scavengers of free radicals, and the consequent implication in the prevention of the mentioned disorders [35].

Phenolic compounds and other natural products have been implicated in the ability to donate electrons to DPPH that turns from purple to yellow in solution and this reaction can be monitored by spectrophotometry at 518 nm [26]. This assay is widely used to determine antiradical/antioxidant activity of purified compounds and plant extracts. In this aspect, EE, HF, DF, EF, and BF were able to inhibit DPPH-free radical scavenging, demonstrating an expressive antioxidant activity. In particular, EF presented the highest concentrations of total phenolic and flavonoids and was the most active to inhibit the DPPH radical formation. Probably, the flavonoids, including apigenin and luteolin detected in EF (Figure 1), had an important role in the inhibition of free radicals. Therefore, these results suggest that *V. condensata* contains phytochemicals that are capable of donating hydrogen to a free radical in order to remove the odd electron which is responsible for radical's reactivity.

The reducing power of iron is used to measure the reductive ability of antioxidant through the transformation of Fe^+3^ to Fe^+2^ monitored by absorbance measurement at 700 nm [36]. This reducing property has been shown to exert antioxidant action by donate a hydrogen atom to break the free radical chain [28]. EE, HF, DF, EF, and BF transformed Fe^+3^ to Fe^+2^, demonstrating a reducing potential of *V. condensata*. This data confirmed the previous findings observed by DPPH method described in details above. The ability to reduce Fe^+3^ may be attributed to the hydrogen donation from the phenolic compounds [37], which is also related to the presence of the reductant agent [38]. In addition, the number and position of hydroxyl group of the phenolic compounds also regulate this antioxidant activity [39].

The obtained results through phosphomolybdenum method demonstrated that DF, EF, and BF exhibited the highest antioxidant capacity for phosphomolybdate reduction. The results suggested that the strong antioxidant activity of extracts might be due to the presence of phenolics compounds present in the extract [40]. Moreover, the flavonoids apigenin and luteolin detected in the present investigation may contribute to the phosphomolybdate scavenging activity [41].

MDA, a major degradation product of lipid hydroperoxides, is more attractive as a marker for assessing the extent of lipid peroxidation [42, 43]. This compound showed to be mutagenic and carcinogenic and was implicated in some pathological processes [43]. MDA may be determined by TBA method [44]. Thus, the results obtained in the present investigation demonstrated that EE, HF, DF, EF, and BF of *V. condensata* reduced the production of MDA (Table 3). However, after 5 days of the experiment, EF was more effective to inhibit the formation of MDA, demonstrating the great potential in the search for new compounds with antioxidant activity.

It was the first time that the quantification of the total phenolic and flavonoid contents of EE, HF, DF, EF, and BF of *V. condensata* leaves was reported in the literature. EF showed the highest total phenolic and flavonoid contents. Phenolic constituents, among them the flavonoids, have been investigated for antioxidant [45], antimicrobial [46], antinociceptive, and anti-inflammatory properties [47, 48]. In addition, flavonoids have been isolated and identified in different species of *Vernonia*, which may represent an important chemotaxonomic marker [34].

Considering the phenolic constituents present in *Vernonia* species, studies with *V. amygdalina* showed that the content of total phenols varied in accordance with references as follow: 0.20 to 0.27 g/100 g [49], from 0.061 to 1.11 g/100 g [50], and 0.24 g/100 g [51]. In *Vernonia blumeoides*, the content of total phenols ranged from 0.014 to 0.41 g/100 g in ethanol, chloroform, ethyl acetate, and butanol extracts [52]. In solvents of different polarities, the present results revealed a total phenolic content ranging from 0.19 to 23.11 g/100 g demonstrating that *V. condensata* is an excellent source of phenolic substances (Table 1). Based on the physicochemical characteristics, EF was more effective to extract the total phenols, which was in agreement with previous studies of *Vernonia*'s species [50, 52].

In *V. amygdalina*, the total flavonoid content, another important phenolic parameter, was 0.22 g/100 g [51] or 0.041 to 0.466 g/100 g [50] depending on the author. The results presented in Table 4 showed that, using solvents of different polarities, the flavonoid content ranged from 0.13 to 4.10 g/100 g indicating that *V. condensata* is a promising source of this compound. As the quantification of total phenols, ethyl acetate produced a higher yield of flavonoids and this solvent could be the most appropriate to extract substances of this class of special metabolite in *Vernonia* genus.

In view of the chemical studies on the bioactive components of this genus, among the identified constituents, flavonoids that exhibit antioxidant activity are highlighted [18, 33, 48]. A simple, rapid, and accurate High-Performance Liquid Chromatographic (HPLC) method was developed to identify these compounds in *V. condensata*. Two flavonoids, apigenin [53] and luteolin [34], were selected since these substances could explain the antioxidant action of *Vernonia*. However, besides flavonoids, other phenolic compounds such as condensed and hydrolysable tannins, not identified in the present study, could also contribute with the antioxidative effectivity of *V. condensata* [7].

Evidence of natural products with antioxidant activity has been described in the literature and this property seems to add up to that of the endogenous antioxidant system [54]. In the last years, studies focused on medicinal plants with antioxidant potential have been developed [55, 56]. These researches arise from the formation of special metabolites that are produced by plants to protect them from free radicals generated by oxidative stress imposed by solar radiation and other environmental stresses [57]. Among the phenolic constituents present in plant extracts that can be highlighted the flavonoids, tannins, catechins, proanthocyanidins, and some polyphenolic acids [55, 58].

## 5. Conclusion

Based on the results obtained in the present study, the presence of antioxidant effect of the ethanol extract and fractions obtained from *V. condensata* leaves can be stated in the occurrence of substances that present donor of electrons and protons capable of neutralizing free radicals and make them more stable products plus the ability to reduce oxidative intermediates of lipid peroxidation processes. The total phenolic and flavonoid contents were also determined to quantify and identify antioxidant constituents and EF showed the highest values established. Finally, the chemical characterization revealed the presence of apigenin and luteolin as previously reported in other species of the genus *Vernonia*. All this data sustain that *V. condensata* is an important and promising source of bioactive substances with antioxidant activity.

## Figures and Tables

**Table 1:** Antioxidant activity of the ethanol extract and fractions obtained from *Vernonia  condensata *leaves by DPPH and Fe^+3^ reducing power methods.

Plant extract/chemical	IC_50_ (*μ*g/mL)	
	DPPH	Fe^+3^ Reducing power
Ethanol extract (EE)	35.44 ± 0.76	54.42 ± 0.19
Hexane fraction (HF)	147.14 ± 0.40	212.45 ± 0.02
Dichloromethane fraction (DF)	51.69 ± 0.44	336.48 ± 11.05
Ethyl acetate fraction (EF)	18.44 ± 0.54	19.98 ± 0.42
Butanol fraction (BF)	48.45 ± 0.26	53.88 ± 0.08
Rutin	3.00 ± 1.80	—
Ascorbic acid	—	1.73 ± 0.04

Each value in the table is represented as mean ± S.E.M. (*n* = 3). The values are significantly different (*P* < 0.05).

**Table 2:** Antioxidant activity of the ethanol extract and fractions from *Vernonia condensata *leaves by phosphomolybdenum reducing power method.

Plant extract/chemical	Relative antioxidant activity (%)	
	Rutin	Ascorbic acid
Ethanol extract (EE)	94.87 ± 0.09	24.90 ± 0.02
Hexane fraction (HF)	56.02 ± 0.06	13.54 ± 0.01
Dichloromethane fraction (DF)	135.00 ± 0.04	32.63 ± 0.01
Ethyl acetate fraction (EF)	127.00 ± 0.01	30.68 ± 0.01
Butanol fraction (BF)	105.00 ± 0.04	25.34 ± 0.01

Each value in the table is represented as mean ± S.E.M. (*n* = 3). The values are significantly different (*P* < 0.05).

**Table 3:** Concentration of MDA in mmol/L in the TBA test with ethanol extract and fractions from *Vernonia  condensata* leaves.

Plant extract/chemical	Concentration (mg/mL)	MDA concentration (mmol/L)				
		Day 0	Day 1	Day 2	Day 3	Day 4
Control	—	0.29 ± 0.01^A^	0.39 ± 0.05^A^	0.46 ± 0.02^A^	0.95 ± 0.01	1.33 ± 0.01

BHT	7.5	0.43 ± 0.01	0.68 ± 0.03	0.60 ± 0.06	0.18 ± 0.01	0.20 ± 0.01
	15	0.17 ± 0.02	0.43 ± 0.02^A^	0.36 ± 0.01^A^	0.17 ± 0.01	0.17 ± 0.01
	30	0.18 ± 0.01^A^	0.18 ± 0.01	0.20 ± 0.01	0.15 ± 0.01	0.14 ± 0.01

Ethanol extract (EE)	7.5	0.61 ± 0.01	0.32 ± 0.01^A^	0.67 ± 0.01	0.49 ± 0.01	0.45 ± 0.01
	15	1.04 ± 0.01	0.56 ± 0.03	0.40 ± 0.01^A^	0.48 ± 0.02	0.46 ± 0.01
	30	0.57 ± 0.07	0.68 ± 0.01	0.25 ± 0.01	0.42 ± 0.04	0.43 ± 0.03

Hexane fraction (HF)	7.5	0.17 ± 0.01	0.42 ± 0.01^A^	0.48 ± 0.01^A^	0.68 ± 0.03	0.71 ± 0.04
	15	0.38 ± 0.01^A^	0.43 ± 0.03^A^	0.47 ± 0.01^A^	0.55 ± 0.01	0.65 ± 0.01
	30	0.32 ± 0.04^A^	0.56 ± 0.01	0.46 ± 0.01^A^	0.61 ± 0.01	0.73 ± 0.01

Dichloromethane fraction (DF)	7.5	0.18 ± 0.01	0.21 ± 0.01	0.37 ± 0.01	0.39 ± 0.01	0.35 ± 0.01
	15	0.21 ± 0.01^A^	0.20 ± 0.01	0.44 ± 0.01^A^	0.42 ± 0.01	0.27 ± 0.01
	30	0.28 ± 0.01^A^	0.20 ± 0.07	0.38 ± 0.01^A^	0.36 ± 0.00	0.38 ± 0.01

Ethyl acetate fraction (EF)	7.5	0.26 ± 0.01^A^	0.39 ± 0.01^A^	0.59 ± 0.01	0.18 ± 0.01	0.54 ± 0.01
	15	0.26 ± 0.01	0.25 ± 0.01^A^	0.37 ± 0.01^A^	0.35 ± 0.01	0.34 ± 0.01
	30	0.28 ± 0.01^A^	0.28 ± 0.01	0.20 ± 0.01	0.19 ± 0.01	0.18 ± 0.01

Butanol fraction (BF)	7.5	0.33 ± 0.01	0.75 ± 0.01^A^	0.46 ± 0.02^A^	0.57 ± 0.01	0.38 ± 0.01
	15	0.34 ± 0.01^A^	1.21 ± 0.01	0.57 ± 0.01	0.69 ± 0.01	0.36 ± 0.03
	30	0.17 ± 0.01	0.64 ± 0.01	0.52 ± 0.01^A^	0.64 ± 0.01	0.42 ± 0.01

Each value in the table is represented as mean ± S.E.M. (*n* = 3). Capital letter (A) in the same column, means do not differ (*P* < 0.05) when compared to the control group.

**Table 4:** Total phenolic and flavonoids contents obtained with ethanol extract and fractions from *Vernonia condensata* leaves.

Plant extract	Total phenols (g/100 g)	Total flavonoids (g/100 g)
Ethanol extract (EE)	11.73 ± 0.18	0.16 ± 0.01
Hexane fraction (HF)	0.19 ± 0.03	0.13 ± 0.01
Dichloromethane fraction (DF)	2.48 ± 0.08	1.48 ± 0.02
Ethyl acetate fraction (EF)	23.11 ± 0.90	4.10 ± 0.03
Butanol fraction (BF)	15.14 ± 0.07	0.94 ± 0.00

Each value in the table is represented as mean ± S.E.M. (*n* = 3). The values are significantly different (*P* < 0.05).
